# Stable Encoding of Visual Cues in the Mouse Retrosplenial Cortex

**DOI:** 10.1093/cercor/bhaa030

**Published:** 2020-03-07

**Authors:** Anna Powell, William M Connelly, Asta Vasalauskaite, Andrew J D Nelson, Seralynne D Vann, John P Aggleton, Frank Sengpiel, Adam Ranson

**Affiliations:** 1 School of Psychology, Cardiff University, CF10 3AS Cardiff, UK; 2 School of Medicine, University of Tasmania, Hobart, TAS, Australia; 3 School of Biosciences, Cardiff University, Cardiff, CF10 3AX, UK; 4 Neuroscience and Mental Health Research Institute, Cardiff University, Cardiff, CF24 4HQ, UK; 5 Faculty of Medicine and Health Sciences, Department of Basic Sciences, Universitat Internacional de Catalunya, Barcelona, 08195, Spain; 6 Institut de Neurociènces, Universitat Autònoma de Barcelona, Bellaterra, 08193, Spain

**Keywords:** behavior, navigation, retrosplenial cortex, V1, vision

## Abstract

The rodent retrosplenial cortex (RSC) functions as an integrative hub for sensory and motor signals, serving roles in both navigation and memory. While RSC is reciprocally connected with the sensory cortex, the form in which sensory information is represented in the RSC and how it interacts with motor feedback is unclear and likely to be critical to computations involved in navigation such as path integration. Here, we used 2-photon cellular imaging of neural activity of putative excitatory (CaMKII expressing) and inhibitory (parvalbumin expressing) neurons to measure visual and locomotion evoked activity in RSC and compare it to primary visual cortex (V1). We observed stimulus position and orientation tuning, and a retinotopic organization. Locomotion modulation of activity of single neurons, both in darkness and light, was more pronounced in RSC than V1, and while locomotion modulation was strongest in RSC parvalbumin-positive neurons, visual-locomotion integration was found to be more supralinear in CaMKII neurons. Longitudinal measurements showed that response properties were stably maintained over many weeks. These data provide evidence for stable representations of visual cues in RSC that are spatially selective. These may provide sensory data to contribute to the formation of memories of spatial information.

## Introduction

The rodent retrosplenial cortex (RSC), which comprises much of the medial part of the dorsal cortex, is reciprocally connected to the hippocampal formation, anterior thalamic nuclei, and visual cortex. It is, therefore, perhaps unsurprising that this cortical region has been repeatedly shown to be important for spatial memory and navigation (Vann et al. 2009). However, there is still much uncertainty with respect to the nature of the RSC’s contribution to these processes. One proposal, which has been at the forefront of RSC research for the last decade, is that this structure has an important role in integrating sensory and nonsensory information (Wolbers and Büchel 2005; Byrne et al. 2007; Vann et al. 2009; Alexander and Nitz 2015); this proposal reflects its anatomical connectivity, making it well placed to combine cortical and subcortical sensory and motor-related signals. Consistent with a role in integrating sensory signals, experiments in anesthetized mice provide evidence of sensory responses in the RSC (Murakami et al. 2015; Zhuang et al. 2017), while other work has described visually evoked responses in feedback axons relaying signals between the RSC and visual cortex (Makino and Komiyama 2015). Meanwhile, unit recordings in freely moving rats have showing that retrosplenial unit ensembles simultaneously map position in both the external and internal frames of reference (Alexander and Nitz 2015).

This integrative model of retrosplenial function is further supported by behavioral data, as RSC lesioned rats are particularly impaired on tasks where they are explicitly called upon to integrate different types of information (Cooper and Mizumori 1999; Vann and Aggleton 2002; Pothuizen et al. 2008; Elduayen and Save 2014; Hindley et al. 2014). Despite the apparent importance of the RSC for spatial cognition, there is surprisingly little evidence from awake animals examining how sensory information required for these functions are encoded in the RSC. In particular, the extent to which simple visual stimuli are encoded in an abstract or higher order versus veridical representation (i.e., which encodes the physical properties of the stimulus) remains unclear. More broadly, it remains uncertain how visual drive is integrated with motor input in the RSC. Finally, it remains unknown to which extent RSC visual and nonvisual representations, in individual neurons, are longitudinally stable (as in early sensory cortex) or alternatively dynamic and context sensitive.

To address this gap in our knowledge, we used cellular resolution 2-photon imaging, together with genetic labeling of cell types, to assess how visual and motor-related signals are organized and processed in the RSC. A key step was to study head-fixed awake animals, allowing precise control and disentanglement of visually driven and motor-driven neural activity. This allowed an examination of whether overlapping or distinct populations of RSC neurons process visual and motor signals, and how stable or dynamic representations of these signals are in single neurons over timescales of weeks.

## Materials and Methods

### Animals

All experimental procedures were carried out in accordance with the UK Animals (Scientific Procedures) Act 1986 and European Commission directive 2010/63/EU. Mice expressing GCaMP6f were generated by crossing the Ai95D line (Jax, 024105) with either the CaMKII-alpha-cre T29-1 line (Jax, 005359), for expression in CaMKII positive cells, or the PV-cre line (Jax, 008069), for expression in parvalbumin (PV) positive cells. Experiments were carried out on adult male mice, housed under normal light conditions (12:12 h light:dark). All recordings were made during the light period.

### Animal Surgical Preparation

All surgical procedures were conducted under aseptic conditions. Prior to cranial window surgery, animals were administered with the antibiotic Baytril (5 mg/kg, s.c.) and the anti-inflammatory drugs Rimadyl (5 mg/kg, s.c.) and dexamethasone (0.15 mg/kg, i.m.). Anesthesia was induced at a concentration of 4% isoflurane in oxygen and maintained at 1.5–2% for the duration of the surgery. Once anesthetized, animals were secured in a stereotaxic frame (David Kopf Instruments) and the scalp and periosteum were removed from the dorsal surface of the skull. For RSC recordings, a custom head plate was attached to the cranium using dental cement (Super Bond, C&B), with an aperture approximately centered over the right hemisphere RSC. A 3-mm circular craniotomy was then made, centered approximately 2.5 mm caudal to bregma. The lateral placement of the craniotomy was such that it encompassed a small portion of the left hemisphere. This positioning meant that is was possible to visualize the central sinus through the cranial window, which provided a useful reference point when imaging. The craniotomy was closed with a glass insert made from three layers of circular glass (#1 thickness; 1 × 5 mm, 2 × 3 mm diameter) bonded together with optical adhesive (Norland Products; catalogue no. 7106). The window was placed such that the smaller pieces of glass were in contact with the brain surface and the larger piece rested on the skull surrounding the craniotomy. The window was then sealed with dental cement. After surgery, all animals were allowed at least 1 week to recover before being imaged. For V1 recordings, the procedure was performed as described above except the craniotomy and head plate position was centered −3.4 mm posterior and 2.8 mm lateral from bregma of the right hemisphere.

### Imaging and Locomotor Data Acquisition

In vivo 2-photon imaging was performed using a resonant scanning microscope (Thorlabs, B-Scope) with a 16× 0.8NA objective (Nikon). GCaMP6 was excited at 980 nm using a Ti:sapphire laser (Coherent, Chameleon) with a maximum laser power at sample of 50 mW. Data were acquired at approximately 60 Hz and averaged, resulting in a frame rate of approximately 10 Hz. For all imaging experiments, animals were head-fixed and free to run on a custom designed fixed-axis cylindrical treadmill. Movement was measured using a rotary encoder (Kübler, 05.2400.1122.0100). Imaging, behavioral, and visual stimulation timing data were acquired using custom written DAQ code (Matlab) and a DAQ card (NI PCIe-6323, National Instruments).

### Visual Stimuli

Visual stimuli were generated in Matlab using the psychophysics toolbox (32), and displayed on two calibrated LCD screens (Iiyama, B2080HS; width × height 26 × 47 cm) at right angles to one another placed 20 cm from the eye. All visual stimuli were circular, unidirectionally drifting square wave gratings of uniform size (40 × 40 °), spatial (0.08 cycles per degrees) and temporal frequency (1 Hz) presented at full contrast. Stimulus position was corrected for viewing angle. Stimulus parameters were optimized in pilot experiments. Orientation and spatial location varied depending on the experiment. For all experiments, drifting gratings were presented for 2 s followed by a 2-s intertrial interval during which a gray screen was displayed. In all experiments, visual stimuli were presented passively, such that the drifting of the grating was not yoked to the animal’s movement.

### Experimental Design

Low magnification imaging of the entire extent of RSC visible within the cranial window was used to localize the visually responsive region. In order to elicit visually driven activity, a vertically orientated drifting grating was presented in the center of the binocular region of the visual field, centered at 20 ° elevation. Fro analysis of visual responsiveness as a function of depth, Recordings were made at 11 different depths from pial surface to −300 μm, at two partially overlapping 900 × 900 μm fields of views within the RSC (one rostral and one caudal).

For experiments to determine spatial and orientation selectivity, 48 different stimuli were presented, with each stimulus repeated 10 times in a pseudorandom order. The stimuli were circular drifting gratings of one of eight different orientations, presented in one of six locations in a 3 × 2 grid, centered at 0, 45, and 90 ° in azimuth, and 0 and 20 ° in elevation. Recordings were made from a 400 × 400 μm field of view, aligned on the medial side with the midline, and centered approximately 1.75 and 3.25 mm caudal of bregma for the rostral and caudal recording sites, respectively. Recordings were made at depths of 150–250 μm.

### Calcium Imaging Data Analysis

Calcium imaging data were registered and segregated into neuronal regions of interest (ROI) using Suite2P (Pachitariu et al. 2016). Pixel-wise stimulus preference maps were constructed by first calculating the mean of the registered imaging frames recorded during the drifting phase of each stimulus, and then determining for each pixel, the stimulus which elicited the largest mean response.

For experiments in which the stability of visual responses was tracked longitudinally, recordings were made from the same field of view over multiple sessions. Cortical surface vascular landmarks were used to locate the same neurons between sessions. In order to match ROI masks detected with Suite2P between imaging sessions, the cpselect, fitgeotrans, and imwarp functions in Matlab were used to warp the ROI masks using manually identified control points selected from the mean image frame from each experiment. Masks that overlapped by more than 60% were detected in all sessions and were flagged as potential longitudinally recorded neurons. These masks were then manually verified by visual observation of the region of the mean each frame that they corresponded to in each experiment.

## Quantification and Statistical Analysis of Visual and Motor Responses

The visual responses of individual neurons were quantified as the mean d*F*/*F* value between 0.5–1.5 s after stimulus onset, with a baseline period quantified for each trial as the mean d*F*/*F* between 1 and 0 s before stimulus onset. Stimulus response was fit with the product of a 2D Gaussian (to represent the spatial extent of the receptive field) and the sum of two Von Mises functions (to represent the orientation selectivity of the neuron). Specifically, let

$$ {A}_{\left(x,y\right)}=a\times e\frac{-{\left(x-{\mu}_x\right)}^2}{2{\sigma}_x^2}+\frac{-{\left(y-{\mu}_y\right)}^2}{2{\sigma}_y^2} $$

where *a* is the maximum response, *x* is the position of the stimuli, μ*_x_* is the center of the receptive field, and σ*_x_* is the spatial extent of the receptive field, respectively, in the horizontal dimension. Similarly, *y* is the position of the stimuli, μ*_y_* is the center of the receptive field, and σ*_x_* is the spatial extent of the receptive field, respectively, in the vertical dimension.

Furthermore, let

$$ M\left(\theta \right)=e\left(\kappa \kern0.5em \cos \left(\theta -{\theta}_{\mathrm{Pref}}\right)\right)+\xi \times e\left(\kappa \kern0.5em \cos \left(\theta -{\theta}_{\mathrm{Pref}}-\pi \right)\right) $$

where κ is the dispersion of the response and is related to the tuning width of the neuron. θ is the orientation of the stimuli and θ_Pref_ is the orientation preference of the neuron. Finally, ξ is the ratio of the response when the stimulus is displayed at θ_Pref_ to the response at θ_Pref_–π radians.

The overall response of a neuron to stimuli of different positions and orientations was fit with:

$$ {\mathrm{Response}}_{\left(x,y.\theta \right)}={A}_{\left(x,y\right)}\times{M}_{\left(\theta \right)}+E $$

where *Ε* is the background firing rate.

Our experience was that Θ_Pref_ was difficult to fit due to numerous local minima, hence the model was repeatedly fit with Θ_Pref_ starting at all presented orientations, and the fit with the lowest residual was chosen as the best fit. This model was initially fit with the *lsqcurvefit()* function and subsequently, model performance was evaluated with *fitnlm()* and we excluded from further analysis fits where *R*^2^ < 0.5. Cross sections of the fits of the neurons included for further analysis were manually inspected and compared with the raw data (i.e., the orientation tuning curve at the fitted preferred position, and the 2D Gaussian at the fitted preferred orientation). Supplementary Figure S8 shows examples of raw data and cross sections of representative fits of varying goodness of fit.

Orientation tuning curves were calculated using data pooled over the two directions of grating drift. Orientation tuning fit curves were used to calculate orientation selectivity index (OSI) defined as (peak response−trough response)/peak response.

An integration index was calculated to quantify the linearity of integration of visually and locomotion evoked activity in RSC neurons. The integration index was calculated as: (integrated − sum of isolated)/(integrated + sum of isolated). Here “integrated” is defined as the mean response of a single neuron during simultaneous visual stimulation at its preferred orientation and any form of locomotion, and “sum of isolated” is defined as the sum of the response during locomotion without visual stimulation and visual stimulation without locomotion.

A neuron was classified as visually responsive if it showed a statistically significant increase in activity between the baseline and visual stimulation periods for at least one stimulus, with statistical significance calculated with a shuffle test (1000 shuffles, *P* < 0.05), corrected for multiple comparisons using the mafdr false discovery rate function in Matlab. Note that, using these criteria approximately 5% of neurons would be expected to be classified as visually responsive by chance. A one-way ANOVA of response amplitudes over stimulus position, orientation, or direction was used to assess whether neurons discriminated these stimulus features. Statistical significance of correlations between run speed and neural activity was calculated with a shuffle test in which the entire trace of run speed was randomly shifted relative to neural activity traces 1000 times (*P* < 0.05). Where appropriate similarity of variance and normality of distribution were checked with the vartestn Matlab function, and the Kruskal–Wallis test was used as noted when the assumptions of the one-way ANOVA or *t*-test were not met. *T*-tests used were two sided. Correction of *P* values for multiple comparisons was calculated using the Matlab function multcompare using the Tukey–Kramer method.

**Figure 1:**
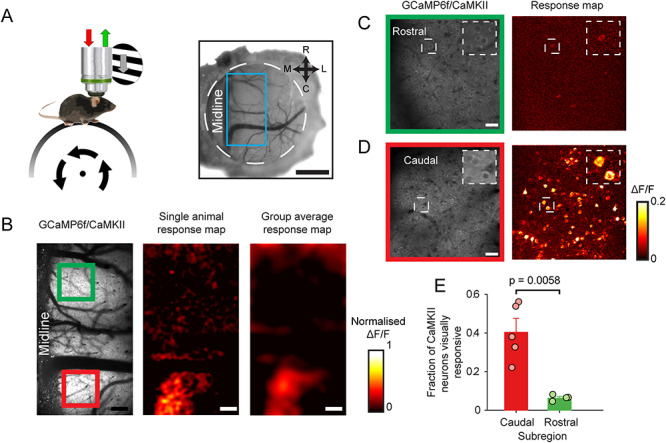
Localization of visually responsive area of dysgranular RSC. (*A*) Setup schematic (left) and cranial window over RSC (right). Blue box indicates area imaged. Scale bar: 1 mm. (*B*) Low magnification 2-photon visual response maps showing visually evoked activity of CaMKII neurons in the cRSC (red box) but not rRSC (green box). Scale bar: 160 μm. (*C*,*D*) Higher magnification of rostral (*C*) and caudal (*D*) regions showing visually responsive neurons in caudal but not rostral areas. Scale bar: 30 μm. (*E*) Mean fraction of visually responsive neurons (error bars represent SEM) detected in caudal and rostral areas.

## Results

### Spatial Organization of Visually Responsive Cells in RSC

We first used low magnification 2-photon imaging of GCaMP6f expressed in CaMKII positive cells (i.e., primarily excitatory neurons, Supplementary Fig. S1*A*) or parvalbumin expressing inhibitory neurons (PV neurons, Supplementary Fig. S1*B*) to localize visually responsive areas of RSC. Recordings were made in awake animals free to walk on a cylindrical treadmill (Fig. 1*A*). We sampled the region between −2.4 and −4.2 mm AP and 0.0 and 0.9 mm ML relative to bregma, and depths between the pial surface and 300 μm. The area was tiled by two partially overlapping 900 × 900 μm fields of view (one anterior, one posterior), and data were acquired at depths spaced by 30 μm in *z* (Supplementary Fig. S1*C*), resulting in 11 depths per field of view. We first generated mean pixel-wise response maps to binocularly presented drifting grating visual stimulation and averaged across the 11 depths. Response maps of the CaMKII RSC population showed a visually responsive area limited to the caudal RSC (cRSC) that was visible both in maps from individual animals and in the mean response maps averaged across five animals (Fig. 1*B*). Analysis of response maps across the 11 depths (spanning 300 μm) showed no systematic variation with depth in the visual responsiveness of this caudal region (Supplementary Fig. S1*D*). Response maps of the parvalbumin retrosplenial cortex (PV RSC) population showed a similar pattern of greater visual responsiveness in the cRSC versus rostral RSC (rRSC), but with significantly lower amplitude (Supplementary Fig. S1*E*). We next made higher resolution recordings from the layer 2/3 of the cRSC and rRSC in which individual CaMKII neurons could be resolved (Fig. 1*C*,*D*). Consistent with our low magnification observations, higher magnification pixel-wise maps showed a large fraction of visually responsive cells in cRSC but not rRSC regions (Fig. 1*E*; see also Supplementary Fig. S2*A*). A cell-wise analysis of visual responses of neurons confirmed that a significantly larger fraction of caudal than rostral neurons were visually responsive (fraction of neurons visually responsive: 40.4 ± 6.4% caudal vs. 6.3 ± 7.4% rostral; *t*-test: *t*(8) = 5.26, *P* = 0.0058; *n* = 5 and *n* = 5 mice, respectively; note that, 5% of neurons are expected to be classified as visually responsive by chance). These results show that visually responsive CaMKII and PV neurons are largely confined to caudal regions of the RSC and that within cRSC a larger fraction of neurons is visually responsive than previously reported in anesthetized animals (Murakami et al. 2015).

**Figure 2:**
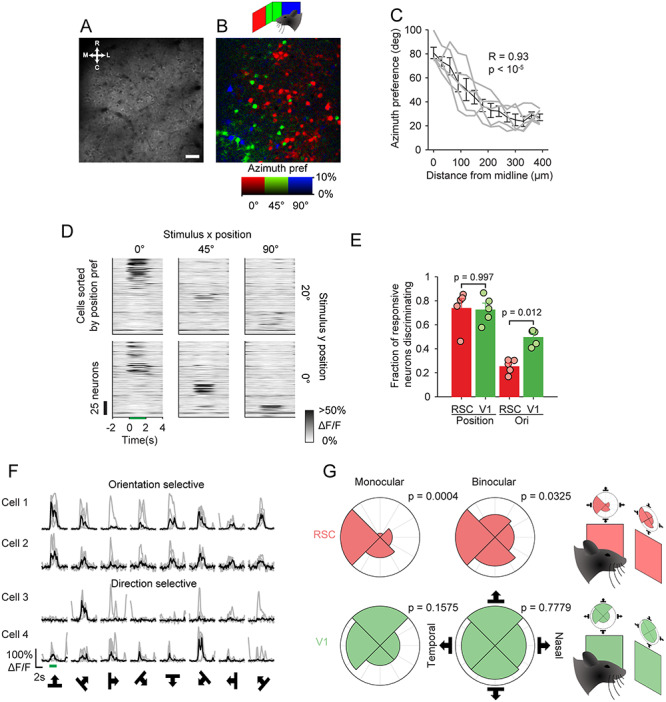
Visual selectivity of CaMKII cRSC neurons. (*A*,*B*) Mean imaging frame from cRSC (*A*), and pixel-wise map from the same field of view of retinotopic preference in azimuth (*B*). Scale bar: 30 μm. (*C*) Azimuth position preference compared with distance of neurons from midline. Gray lines represent individual animals, and black line is average. (*D*) Mean raster plot of neural responses to stimuli at different retinotopic locations, with neurons (rows) sorted by positional preference. (*E*) Mean fractions of neurons (error bars indicate SEM) statistically significantly discriminating stimulus position or orientation in cRSC and V1. (*F*) Example traces of orientation selective and direction selective neurons. (*G*) Polar plots showing distribution of directional preference of RSC neurons (red) and V1 neurons (green) divided into those representing the monocular and binocular visual field. *P* values indicate statistical significance of Raleigh test of nonuniformity and show nonuniformity of RSC direction preference distribution.

**Figure 3:**
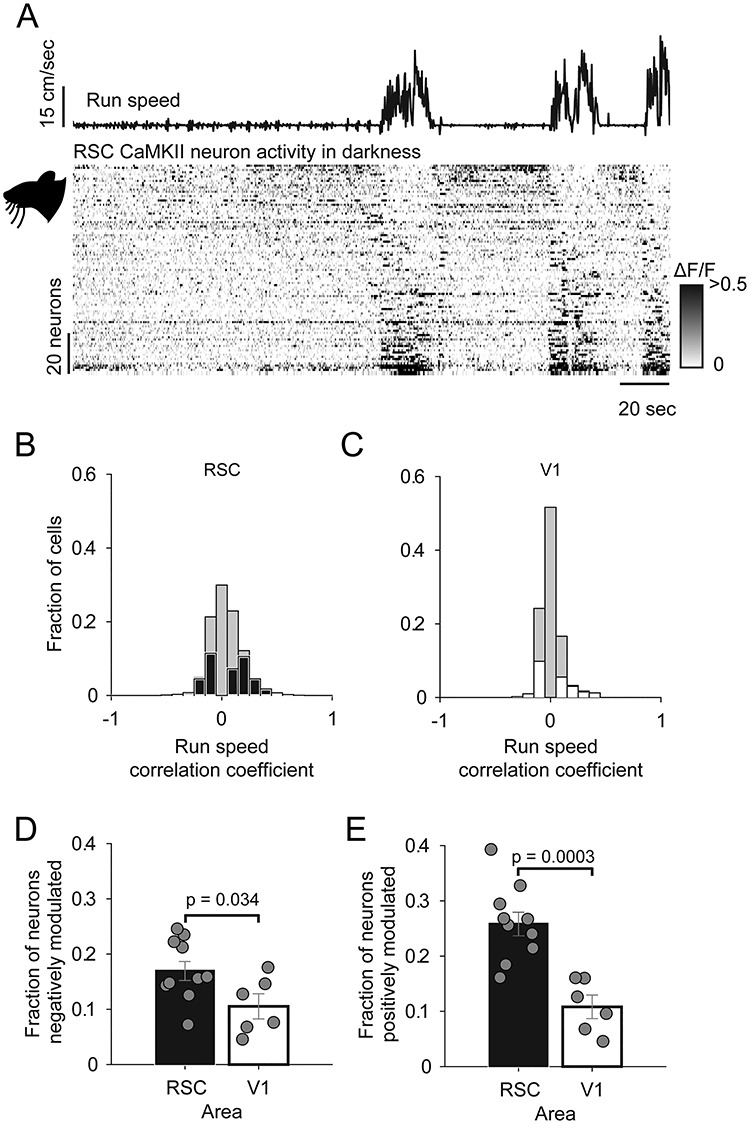
Comparison of the activity of CaMKII RSC and V1 neurons in darkness. (*A*) Run speed, and below, raster representation of neural activity (rows are individual neurons) sorted by run speed correlation. (*B*,*C*) Histogram of run speed correlations of RSC neurons (*B*, black) and V1 neurons (*C*, white) with the nonsignificantly correlated fraction of neurons (determined with shuffle test) represented by gray bars. (*D*,*E*) Mean fraction of neurons which are significantly negatively locomotion correlated (*D*) or positively locomotion correlated (*E*). Error bars indicate SEM.

### Spatial and Orientation Selectivity of Visually Responsive Cells in cRSC

We next investigated the response properties of individual CaMKII neurons in the visually responsive cRSC. A recent study using widefield imaging suggested the presence of a retinotopic map in the RSC (Zhuang et al. 2017). We therefore first tested for the existence of retinotopy at single cell resolution. Animals were visually stimulated with 40 × 40 ° grating patches at one of six retinotopic locations and eight directions (four orientations). Pixel-wise maps were then calculated for azimuth stimulus preference (Fig. 2*B*). This showed a broad range of retinotopic preferences within the 400 × 400 μm field of view, which included the full range of stimulus positions presented. Retinotopic organization was apparent in the pixel maps of azimuth (which we sampled over a broader range than elevation due to restrictions in possible display positions, Fig. 2*B*,*C*; mean correlation between azimuth preference and distance from midline = 0.85 ± 0.07; *n* = 5 mice), and individual neurons typically exhibited spatially selective responses (Fig. 2*D*; Supplementary Fig. S2*B*). In the azimuth map, more medial and lateral parts of the map corresponded to more binocular (i.e., nasal) and monocular (i.e., temporal) visual space, respectively. We next compared the spatial selectivity of cRSC CaMKII neurons to that of CaMKII primary visual cortex (V1) neurons stimulated with the same stimulus. A similar fraction of visually responsive cRSC and V1 neurons discriminated stimulus position (fraction of neurons discriminating stimulus position: 73.7 ± 0.71% cRSC vs. 73.69 ± 7.1% V1; *t*-test: *t*(9) = 0.367, *P* = 0.723; *n* = 5 and *n* = 6 mice respectively; Fig. 2*E*). In contrast, we found that only 25.1 ± 2.6% of visually responsive cRSC neurons significantly discriminated stimulus orientation or direction (at preferred position; Fig. 2*E; F*), which compared with 43.94 ± 0.06% of V1 neurons (*t*-test: *t*(9) = 2.64, *P* = 0.027; *n* = 5 and *n* = 6 mice respectively; Fig. 2*E*). This was reflected in a median orientation selectivity index of 0.63 in cRSC as compared with 0.81 in V1 (Kruskal–Wallis test: *P* < 10^−12^, *n* = 436 and *n* = 307 neurons, respectively). When we binned neurons by their preferred direction of stimulus motion, we additionally observed a significant bias in directional preference of orientation selective cRSC neurons (defined as neurons with OSI > 0.5) toward naso-temporal motion (Fig. 2*G*). This bias was not observed in orientation selective V1 neurons and was apparent both in neurons encoding monocular visual space (Raleigh test for nonuniformity; V1: *n* = 40 neurons; *P* = 0.1575; RSC, *n* = 56 neurons, *P* = 0.0004) and binocular visual space (V1: *n* = 115 neurons, *P* = 0.7779; RSC: *n* = 92 neurons, *P* = 0.0325), although it appeared more pronounced in monocular neurons. Finally, we observed that the degree of habituation of responses over the course of the experiment of visually responsive neurons to their preferred stimuli differed between V1 and RSC neurons, with RSC neurons exhibiting a greater reduction in Δ*F*/*F* than V1 neurons (reduction of 0.35 vs. 0.11; *t*-test: *t*(677) = 3.31, *P* = 0.001; Supplementary Fig. S2*D*). The apparent greater rate of adaptation observed in RSC complicates the interpretation of the findings of fraction of cells responding significantly to different stimulus features. To address this issue, we limited analysis to the first 20% of the trials of the experiment where adaptation is similar in V1 and RSC. While this reduced the overall fractions of discriminating neurons due to the reduction in the number of trials, we observed the same overall pattern described above of similar position discrimination between the RSC and V1, and greater orientation discrimination in V1 (Supplementary Fig. S2*E*). Together these data show that a significant fraction of cRSC neurons shows response selectivity for retinotopy, orientation, and direction of visual flow and as a whole, the population is organized retinotopically.

### Locomotion Sensitivity of RSC Neurons in Darkness

**Figure 4:**
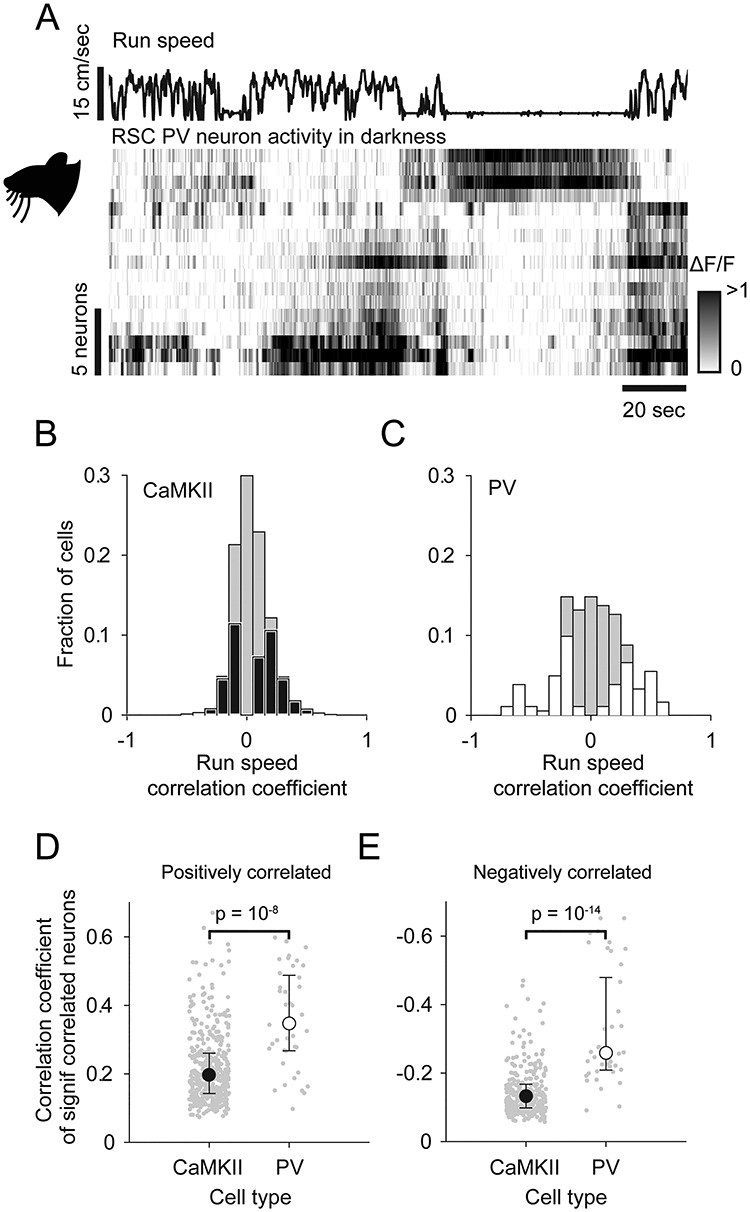
Activity of PV RSC neurons in darkness. (*A*) Run speed, and below, raster representation of neural activity (rows are individual neurons) sorted by run speed correlation. (*B*,*C*) Histogram of run speed correlations of CaMKII neurons (*B*, black) and PV neurons (*C*, white) with the nonsignificantly correlated fraction of neurons represented by gray bars. (*D*,*E*) Median fraction of neurons which is significantly positively locomotion correlated (*D*) or negatively locomotion correlated (*E*). Error bars indicate interquartile range.

Sensory processing by V1 neurons is modulated by the locomotor state of the animal (Niell and Stryker 2010; Keller et al. 2012; Saleem et al. 2013; Ranson 2017; Dipoppa et al. 2018). We next examined the extent to which RSC sensory processing is subject to the same modulation by recording RSC and V1 CaMKII neurons in complete darkness. We found that, in both the RSC and V1, a fraction of neurons was significantly correlated with run speed (either positively or negatively; shuffle test, *P* < 0.05; Fig. 3*A*–*C*; see Supplementary Fig. S3*C* for a similar plot from an example V1 recording). Both cRSC and rRSC exhibited a similar pattern of run speed correlations in darkness and so were combined in this analysis (see Supplementary Fig. S3*A*,*B* for data split into rostral and caudal regions). A larger fraction of RSC than V1 CaMKII neurons was found to be significantly positively correlated with run speed (25.9 ± 0.02% RSC vs. 10.8 ± 0.02% V1; *t*-test: *t*(9) = 4.32, *P* = 0.0003; *n* = 10 fields of view [five mice] and *n* = 6 fields of view [three mice] respectively, Fig. 3*D*,*E*) or significantly negatively correlated with run speed (17.1 ± 0.02% RSC vs. 10.5 ± 0.02% V1; *t*-test: *t*(9) = 2.43, *P* = 0.0338; *n* = 10 fields of view [five mice] and *n* = 6 fields of view [three mice], Fig. 3*D*,*E*). Consequently, some RSC neurons were found to be active in darkness and reliably suppressed by locomotion (Fig. 3*A*, upper rows), while other neighboring neurons recorded simultaneously exhibited the opposite behavior (Fig. 3*A*, lower rows). We next recorded from PV RSC neurons under the same dark conditions (Fig. 4*A*). A similar distribution was again observed in cRSC and rRSC and so neurons from the two areas were again pooled (see Supplementary Fig. S4*A*,*B* for data split into rostral and caudal regions). In contrast to the CaMKII population (Fig. 4*B*), PV neurons exhibited an overall higher degree of locomotion correlation (Fig. 4*C*), both in neurons which were positively correlated (median *R* = 0.19 CaMKII vs. 0.34 PV; Kruskal–Wallis test *P* < 10^−8^; Fig. 4*B*–*D*) and in negatively correlated neurons (median *R* = −0.13 CaMKII vs. −0.26 PV; Kruskal–Wallis test: *P* < 10^−14^; Fig. 4*B*,*C*,*E*).

### Interaction of Visual and Locomotion Signals in Responses in RSC

**Figure 5:**
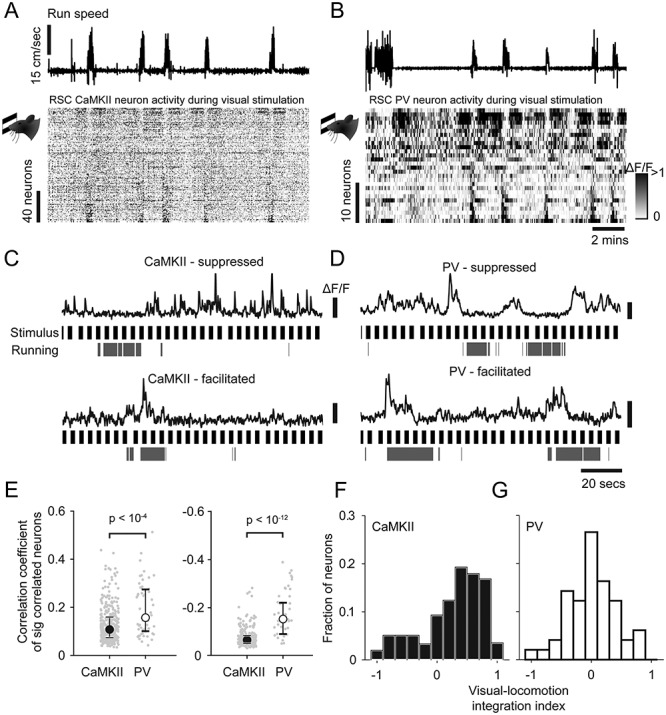
Interaction of visual and locomotion induced activity in CaMKII and PV neurons. (*A*,*B*) Run speed, and below, raster representation of neural activity during visual stimulation sorted by run speed correlation in CaMKII neurons (*A*) and PV neurons (*B*). (*C*,*D*) Example traces of running suppressed (upper) and running facilitated neurons (lower) during visual stimulation, together with running periods and stimulus onset times (black) in CaMKII neurons (*C*) and PV neurons (*D*). (*E*) Median run speed correlations during visual stimulation of significantly positively and negatively correlated neurons (left and right respectively). Error bars indicate interquartile range. (*F*,*G*) Histogram of indices of visual-locomotion integration of CaMKII (black, *F*) or PV (white, *G*) neurons.

We next sought to determine the interaction between visual input and locomotion-related input in individual cRSC neurons (Fig. 5*A*–*D*). We first quantified the extent to which the visually responsive cRSC population overlaps with the locomotion correlated population. To do this, we compared the run speed correlation coefficients of visually responsive versus nonvisually responsive cells. We found no significant differences between these groups, either in CaMKII neurons (Supplementary Fig. S5*A*) or PV neurons (Supplementary Fig. S5*B*), suggesting no correlation between visual stimulus sensitivity and locomotion sensitivity in individual cRSC neurons. As was the case in darkness, we found that PV neurons on average exhibited a higher degree of correlation with run speed than CaMKII neurons during visual stimulation, both in neurons that were positively correlated (median *R* = 0.11 CaMKII vs. 0.16 PV; Kruskal–Wallis test: *P* < 10^−4^; Supplementary Fig. S5*B*, left panel) and in negatively correlated neurons (median *R* = −0.06 CaMKII vs. −0.15 PV; Kruskal–Wallis test: *P* < 10^−12^; Supplementary Fig. S5*B*, right panel). We next studied how visual and locomotion-related activity interacts in RSC neurons, specifically whether the integration of these two types of activity is sublinear, linear, or supralinear. To examine this question, we calculated an integration index which varied between −1 and 1, and where a value of 0 indicates linear integration (i.e., activity when visual stimulation occurs at the same time as locomotion can be explained by the sum of isolated visually and locomotion evoked activity). Negative and positive values of the integration index indicate sublinear and supralinear integration, respectively (see Materials and Methods section). Consistent with supralinear integration of visual and locomotion signals, 76% of CaMKII neurons had an integration index of greater than 0 (*t*-test to test difference of index from zero: *t*(450) = 11.19, *P* < 10^10^), and the CaMKII population overall had a mean integration index of 0.27 ± 0.024. In contrast, on average PV neurons integrated near linearly with an integration index of 0.02 ± 0.058 (*t*-test to test difference of index from zero: *t*(48) = 0.33, *P* = 0.75), and the mean integration indices differed significantly between CaMKII and PV cell types (*t*-test: *t*(498) = 3.31, *P* = 0.001).

In summary, in darkness, RSC neurons are significantly more locomotion modulated than V1 neurons, and within the RSC population, PV neurons are more locomotion modulated than CaMKII neurons, with a subset of PV neurons particularly strongly suppressed during locomotion. Comparison of responses during running and visual stimulation additionally suggests that RSC CaMKII and PV neurons integrate visual- and locomotion-related activity differently: PV responses are largely explained by a summation of activity during visual stimulation and locomotion, while CaMKII neurons exhibit responses that suggest supralinear integration.

### Long-term Stability of RSC Visual and Locomotion Signals

The degree of long-term stability of sensory-evoked activity varies between brain regions (Clopath et al. 2017), with early sensory areas tending to be on the whole more stable (Mank et al. 2008; Margolis et al. 2012; Rose et al. 2016; Ranson 2017; Jeon et al. 2018), while regions containing more abstract representations, such as regions of the hippocampus, exhibit more dynamic and context sensitive representations (Mankin et al. 2012; Ziv et al. 2013). As the RSC occupies an intermediate position between early sensory cortex and the hippocampus, we next examined the extent to which sensory-evoked activity and locomotion modulation of RSC activity was stable over timescales of weeks.

**Figure 6:**
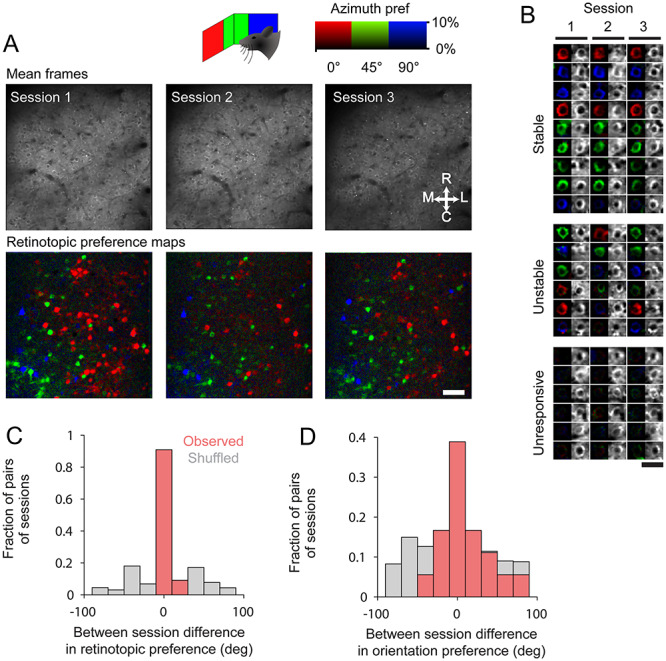
Longitudinal stability of retinotopic positional preference in cRSC neurons over weeks. (*A*) Single field of view imaged over three sessions spread over 4 weeks, showing mean imaging frame (above) and pixel-wise azimuth retinotopic preference map (below). Scale bar: 30 μm. (*B*) Examples of longitudinally tracked neurons, which have stable visual responses, unstable responses or were stably unresponsive. (*C*,*D*) Between session difference in retinotopic preference (*C*) and orientation preferences (*D*) for each neuron on each pair of sessions on which it was measured.

To measure the stability of visually evoked activity, we focused on cRSC and the CaMKII population as this was the area and cell type in which we most frequently observed visually evoked activity. Over the course of 4 weeks, we made three evenly spaced recordings of the same CaMKII cRSC neurons, relocated from session to session using vascular landmarks. Pixel-wise maps of stimulus preference showed clear similarity of retinotopic preference in all animals measured over the 4-week period (Fig. 6*A*). To quantitatively assess similarity, we segregated the field of view of each experiment into neuronal ROIs and validated each ROI as present and valid in each session. This resulted in 170 unambiguously identified CaMKII neurons from three animals that were visible in all three sessions (mean neurons per animal 56.7 ± 9.9, see Supplementary Fig. S6*A*). We next measured the fraction of neurons with significant visual responses over the three sessions and found that the largest fraction (36.5%) had significant responses in all three sessions with 19.4% responsive in two sessions, 27.7% responsive in one session, and 16.5% of neurons not responding in any session (Fig. 6*B*). For each neuron, we then calculated the difference between its fitted azimuth retinotopic preference for each pair of sessions and, consistent with the observed similarity of pixel maps, found that the majority of neurons (90%) had differences in positional preference of less than 10 ° (mean difference 3.86 ± 0.89, compared with mean difference with shuffled neuron identities 30.91 ± 0.358; Fig. 6*C*). We next compared the similarity of orientation preference in longitudinally identified orientation selective neurons (OSI > 0.5) over all pairs of sessions. The above OSI threshold was used to ensure analysis of stability of orientation preference was only undertaken in neurons in which orientation preference could be accurately measured. We found that while the largest fraction of neurons (38.9%) had orientation preferences which differed by less than 10 ° between sessions, many neurons had large differences in preference (median difference 20.55 °, Fig. 6*D*). This difference in tuning preference is considerably larger than previous reports from awake mouse V1 of median differences in orientation preference of approximately 3–4 ° over a 1-week period (Jeon et al. 2018). In summary, these results show longitudinal stability of visual response tuning over periods of weeks in many cRSC neurons, particularly with respect to retinotopic preference, and to a lesser degree orientation preference.

**Figure 7:**
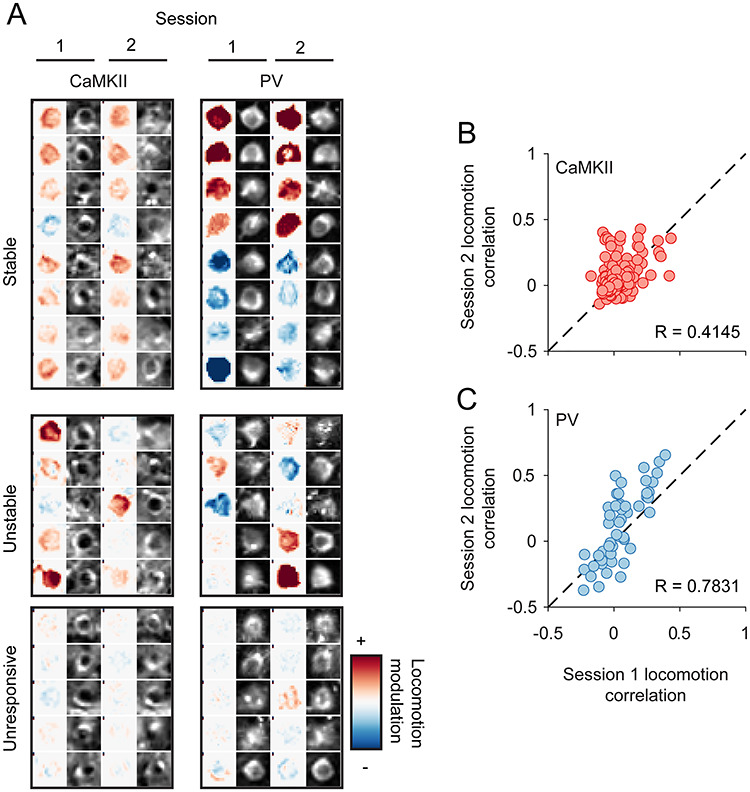
Longitudinal stability of locomotion correlation of cRSC neurons over weeks. (*A*) Examples of longitudinally tracked CaMKII neurons (left) or PC neurons (right) which had stable locomotion modulation, unstable modulation or were stably unmodulated. (*B*,*C*) Inter-session comparison of locomotion modulation of CaMKII neurons (*B*) or PV neurons (*C*).

We next sought to determine whether the degree of locomotion modulation of individual neurons was also stable over time. As both CaMKII and PV cRSC neurons exhibited clear locomotion modulation, we analyzed both populations with a spacing of 1–2 weeks between recordings. We used the same criteria outlined above to identify PV neurons, which were unambiguously detected in all recording sessions, and this resulted in 52 longitudinally tracked cells from three animals (mean neurons per animal 17.33 ± 1.8; see Supplementary Fig. S7*B*) as well as the 170 CaMKII neurons described above (Supplementary Fig. S7*A*). We generated pixel-wise maps of locomotion modulation by calculating for each pixel (moving frames − stationary frames)/(moving frames + stationary frames) and then segregated this map into the ROIs which could be identified longitudinally (Fig. 7*A*; Supplementary Fig. S7*A*). Neurons of both cell types within cRSC exhibited a range of degrees of locomotion modulation, and a high degree of intersession stability could be seen in many neurons (Fig. 7*A*, columns 1 and 2). We next segregated the field of view into longitudinally tracked neurons and calculated run speed correlations of each neuron in the two sessions (Fig. 7*B*,*C*). This also revealed a striking degree of stability of run speed correlation between sessions that was most pronounced in PV neurons (*R* = 0.78; Pearson’s correlation coefficient: *P* < 10^−11^), but also highly significant in CaMKII neurons (Pearson’s correlation coefficient: *R* = 0.41, *P* < 10^−7^).

In summary, these results show that a large fraction of RSC neurons exhibits a high degree of stability of both sensory responses and modulation by locomotion. PV neurons were observed to exhibit a particularly high correlation with locomotion and also a high degree of stability of run speed correlation over the timescale measured.

## Discussion

The RSC has been suggested to function as an integrative hub for sensory and motor signals. Here, we examined the organization of visually evoked activity within the RSC in awake mice, how it integrates locomotion-related activity, and how stable these motor and visual representations are over periods of weeks.

We observed that a substantial fraction of dysgranular RSC excitatory neurons exhibit visually evoked activity, and that this subpopulation is largely limited to the more caudal RSC. The dysgranular RSC has gradients of connections that are consistent with this observation. In particular, caudal dysgranular RSC is more interconnected (both afferent and efferent) with primary and higher cortical visual areas (Wyss and Van Groen 1992). Consequently, these results suggest that visual responsiveness is concentrated in those RSC areas with direct visual inputs, that is, the intrinsic connections linking different parts of dysgranular RSC (Jones et al. 2005) are not sufficient to convey visual responsiveness as measured in this study. Likewise, it is the caudal dysgranular RSC that shows the clearest difference in c-*fos* activity when contrasting spatial behaviors in the light and the dark (Pothuizen et al. 2009).

We further found that many visually responsive cRSC CaMKII neurons exhibited retinotopic, orientation, and direction selectivity. This was largely preserved over periods of several weeks stably. Unexpectedly, over the short timescale of the daily experimental sessions, we observed an apparent difference between V1 and RSC in the rate of adaptation of visually evoked responses, with RSC adapting more rapidly. This has not previously been reported in other similar studies of visual processing in RSC, which may be due to differences in preparation such as anesthetized versus awake recordings. In future studies of visual responses of RSC, it will be of interest to understand the mechanism of this adaptation, as well as more generally taking potential adaptation into account in experiment design. The observation of a high degree of retinotopic selectivity is perhaps surprising given previous evidence of the importance of dysgranular RSC in tasks dependent on allocentric encoding (Vann and Aggleton 2005) as well as recent evidence of place field-like activity (Mao et al. 2017). Our observation of cellular resolution, retinotopic organization of cRSC is consistent with the view that RSC contains sensory representations of an egocentric nature, by which it is meant that stimuli in specific locations defined by their position relative to the observer can activate specific RSC cells. Therefore, in future work, it will be important to reconcile how the retinotopically structured map of visual space reported here, interacts with any allocentric maps of space. The bias in directional preference of cRSC neurons toward naso-temporal motion may indicate a role in the processing of visual flow information. Computational models show that visual flow provides information about egocentric motion and influences firing patterns in spatially tuned cells during rodent navigation (Raudies et al. 2012). Head direction cells are present in the rodent RSC (Chen et al. 1994), therefore this area may play a role in updating head orientation during movement.

A large number of studies in mice has reported locomotion modulation of both baseline and sensory-evoked cortical activity in early sensory areas, including the primary visual and auditory cortices, with the direction of effects varying between cortical areas, stimulation conditions, and cell types (Niell and Stryker 2010; Ayaz et al. 2013; Polack et al. 2013; Keller et al. 2013; Ranson 2017; Saleem et al. 2013; Benevento et al. 2016; Dipoppa et al. 2018; Shimaoka et al. 2018). Our recordings of RSC in the dark revealed that a significant fraction of dysgranular RSC neurons also exhibit baseline activity, which is either strongly positively or negatively correlated with locomotion. As rRSC is more strongly connected than cRSC to both primary motor and secondary motor cortex (Wyss and Van Groen 1992; Shibata and Naito 2008; Yamawaki et al. 2016), we might have expected to see the strongest correlates of locomotion in rostral regions of RSC. Instead, we observed comparable locomotion-related activity across the rRSC and cRSC subregions, which may indicate that locomotion correlations are driven by other motor- or arousal-related signals such as neuromodulatory input (Fu et al. 2014). These findings may also relate to previous studies showing the importance of the RSC for path integration, which relies on locomotion-derived cues to update internal representations of space (Cooper and Mizumori 1999, 2001; Elduayen and Save 2014).

Interestingly, the visual responsiveness and locomotion modulation of cRSC neurons were found to be largely unrelated to one another, with both highly visual and visually nonresponsive neurons exhibiting similar levels of locomotion modulation on average. The parvalbumin-positive population of inhibitory RSC neurons was particularly strongly modulated by locomotion, both in the dark and during visual stimulation, and this modulation was strikingly stably maintained over the interval of weeks examined. This could provide a mechanism of distinct modes of processing in RSC dependent upon behavioral context, whereby functionally distinct subpopulations are either facilitated or suppressed depending upon locomotion state. Interestingly, PV and CaMKII neurons were found to integrate responses to visual stimulation and responses to locomotion differently. Specifically, PV neurons showed more linear and CaMKII neurons more supralinear response integration on average, suggesting responses of CaMKII neurons could amplify the salience of certain combinations of behavior state and visual input. It may be critical to explore this integrative process in a more enriched context of active navigation to understand the significance of this operation. It is additionally important to note that, we do not know whether this integration is occurring in RSC neurons or elsewhere and being inherited by RSC. In fact, we know that the latter is almost certainly at least partly the case as it is now well established that visually evoked activity in the visual cortex, which provides visual input to RSC, is itself locomotion modulated (Niell and Stryker 2010; Keller et al. 2012; Ayaz et al. 2013; Polack et al. 2013; Saleem et al. 2013; Dipoppa et al. 2018).

Given the diverse roles ascribed to RSC, the similarities to primary visual cortex with respect to the organization of visually responsive neurons and their response properties are surprising. In common with V1, many RSC neurons respond to particular positions, orientations, and directions of flow of visual stimuli, are topographically organized into a retinotopic map, and exhibit stable visual preferences over periods of weeks. It will be of interest to determine how other modalities of sensory information are functionally represented and topographically organized, and how these maps are integrated with those of visually responsive cells.

In summary, our results show that a subpopulation of RSC neurons surprisingly stably relay retinotopic, orientation, and direction information about visual cues as well as locomotion signals. It will be important in future to determine how these view-dependent visual feature representations interact with and contribute to view-independent or allocentric RSC representations. One emergent issue relates to evidence that the RSC may be involved in the identification of stable landmarks in an environment (Auger et al. 2012, 2015; Auger and Maguire 2018; Mitchell et al. 2018). While the separation between view-dependent and view-invariant representations enables different forms of scene and object-based information (Wood 2009), it is their co-operation that would help the formation of landmarks to assist flexible navigation.

## Supplementary Material

Supplementary_bhaa030

## Notes

For the use of GCaMP6S, we acknowledge Vivek Jayaraman, Rex A. Kerr, Douglas S. Kim, Loren L. Looger, Karel Svoboda from the GENIE Project, Janelia Farm Research Campus, Howard Hughes Medical Institute. *Conflict of Interest:* None declared.
