# Supplementary material for: Stable Encoding of Visual Cues in the Mouse Retrosplenial Cortex
Source: Cereb Cortex. 2020 Mar 7;30(8):4424–37. doi: 10.1093/cercor/bhaa030 (PMC7438634; doi:10.1093/cercor/bhaa030)
Supplement: Supplementary_bhaa030 [file supplementary_bhaa030.docx]

Supplementary Material


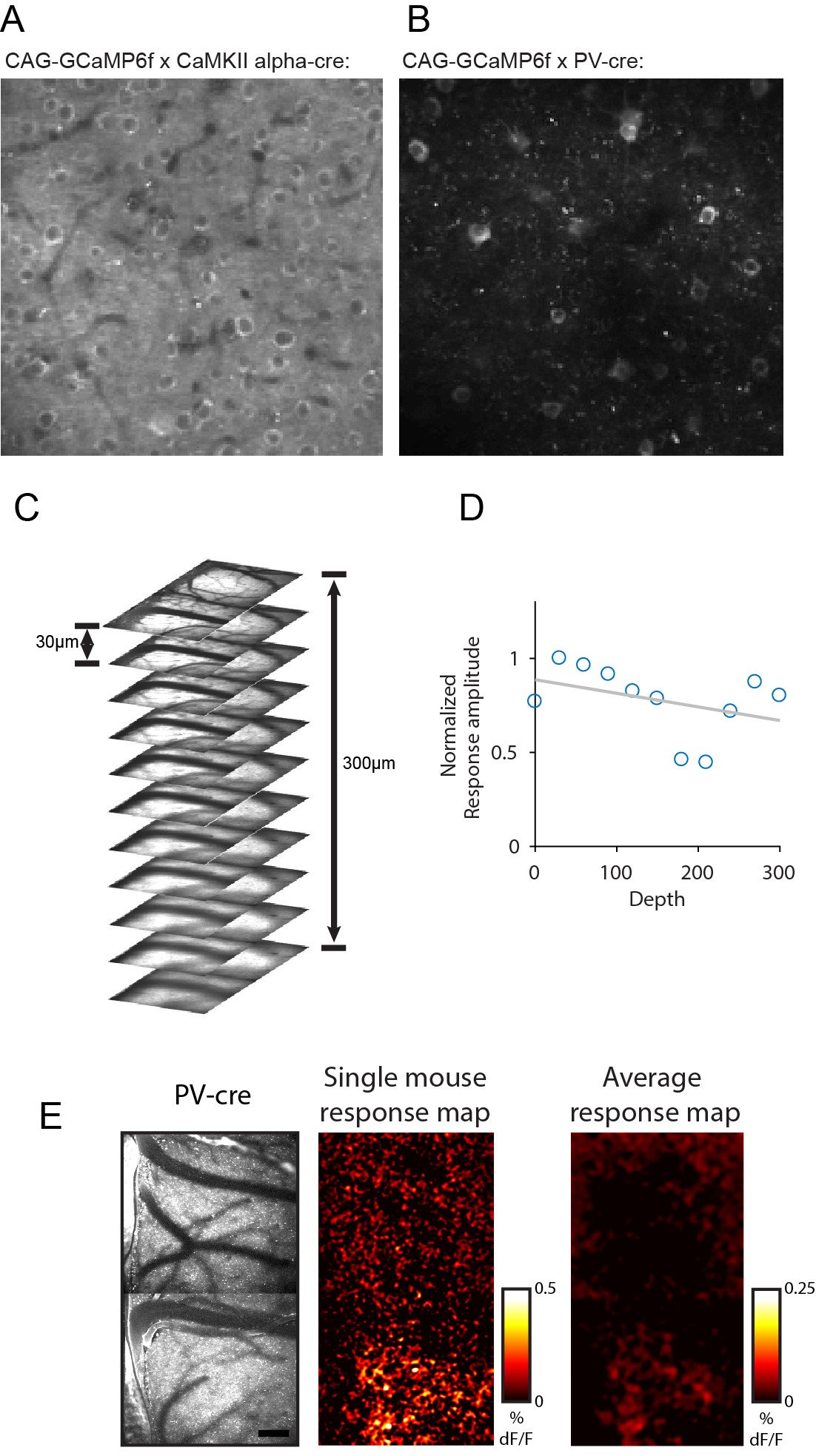


**Figure S1.** **Associated with figure 1.** (A-B) Typical field of view of GCaMP6f labelled neurons obtained from cross of CAG-GCaMP6f x CaMKII-cre mice (A) and from cross of CAG-GCaMP6f x PV-cre mice (B). (C) Typical range of depths collected from each animal during wide field recordings. (D) Normalized response amplitude as a function of depth from surface of brain. (E) Example visual response map from a mouse expressing GCaMP6f in PV neurons and mean response map averaged across 5 mice. Scale bar 160µm.

**
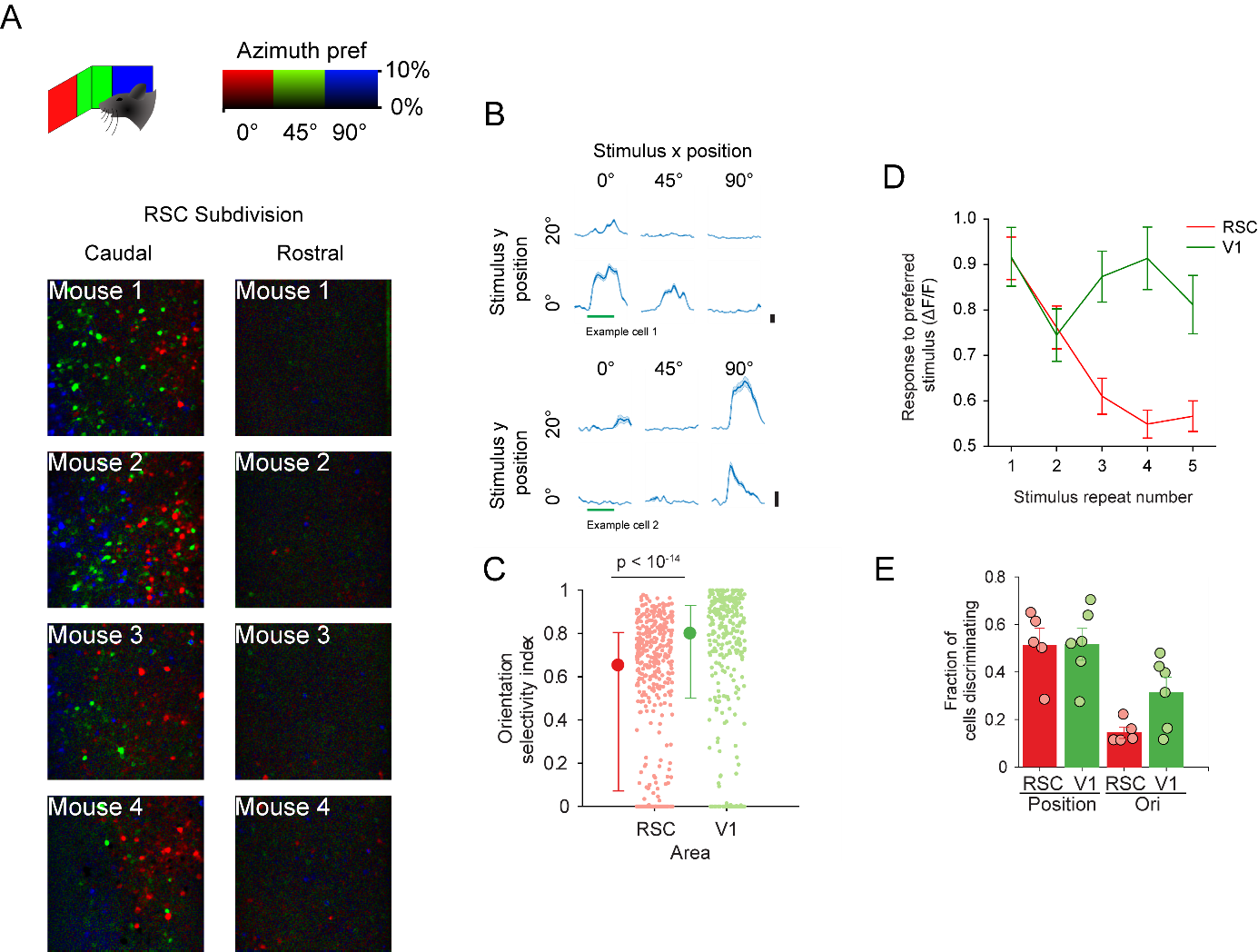
**

**Figure S2.** **Associated with figure 2.** (A) Pixel-wise response maps from 4 mice showing visual responses in caudal (left) but not rostral (right) retrosplenial cortex. (B) Example mean responses of two neurons with distinct spatial tuning, recorded from the same field of view. (C) Distribution of orientation selectivity indices from all recorded CaMKII cRSC neurons. (D) Rate of adaptation of response to preferred stimulus of RSC and V1 neurons. (E) Mean fractions of neurons (error bars indicate S.E.M.) statistically significantly discriminating stimulus position or orientation in cRSC and V1 when only the first 20% of trials were included in analysis (as in Figure 2E).


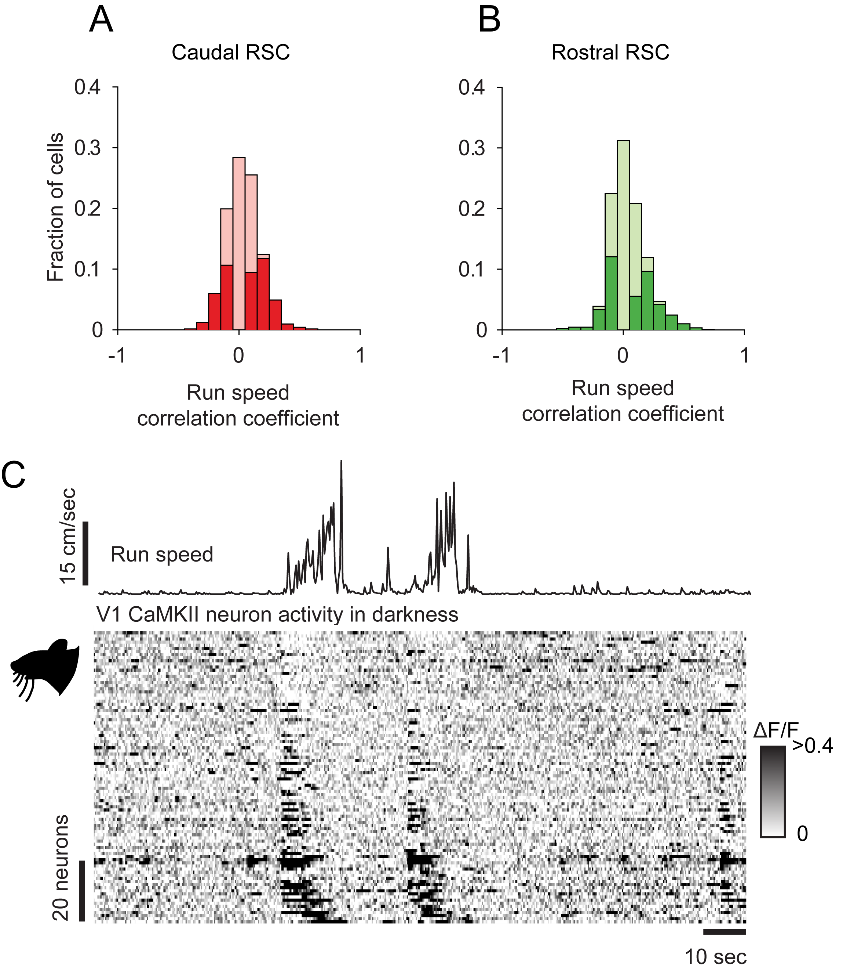


**Figure S3.** **Associated with figure 3.** (A-B) The distribution of run speed correlation coefficients of CaMKII neurons in cRSC and rRSC is similar. (C) Run speed, and below, raster representation of neural activity during visual stimulation sorted by run speed correlation in CaMKII neurons in V1.


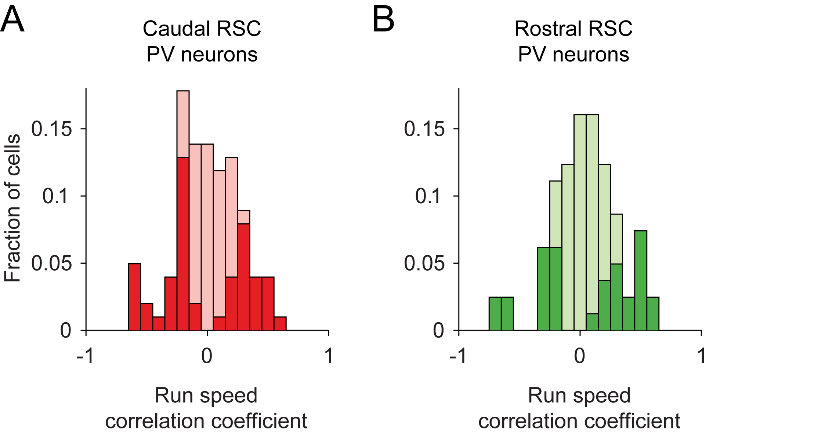


**Figure S4.** **Associated with figure 4.** (A-B) The distribution of run speed correlation coefficients of PV neurons in cRSC and rRSC is similar.


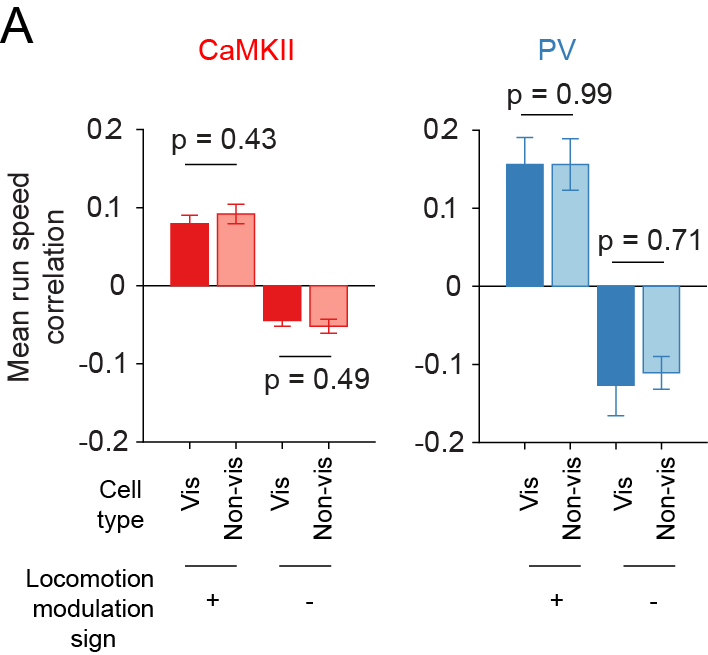


**Figure S5.** **Associated with figure 5.** (A) Visually responsive and non-visually responsive neurons have similar average run speed correlation coefficients.


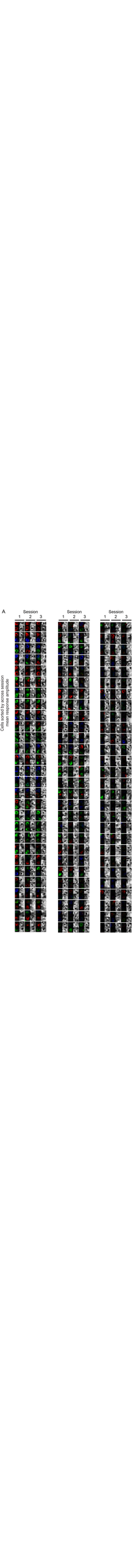


**Figure S6.** **Associated with figure 6.** All manually verified longitudinally imaged cRSC CaMKII neurons, colour coded with azimuth stimulus positional preference.


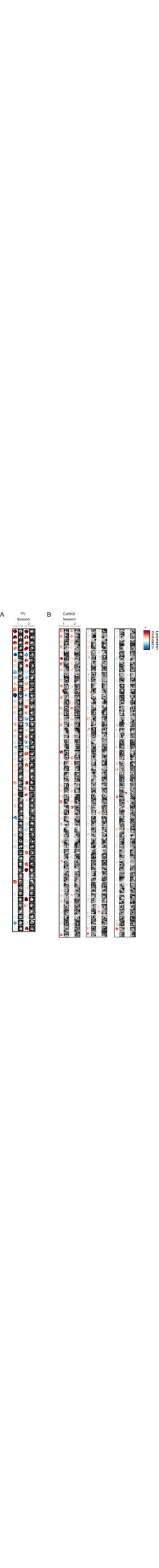


**Figure S7.** **Associated with figure 7.** (A) All manually verified longitudinally imaged cRSC PV neurons colour coded with locomotion modulation, where red indicates positive modulation and blue negative. (B) As in (A), but for cRSC CaMKII neurons


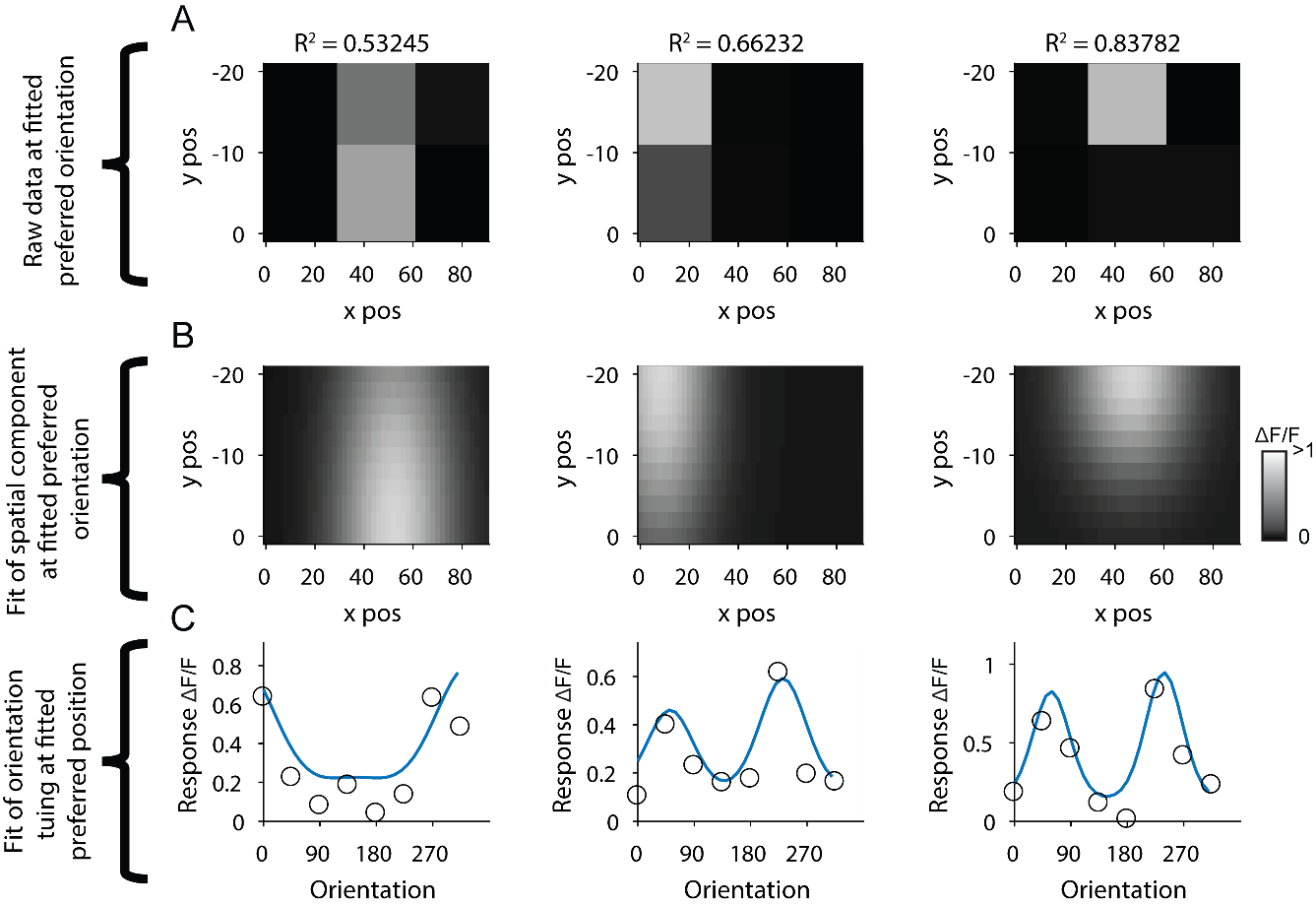


**Figure S8.** (A) Heat plot of representative examples of raw data showing spatial response profiles of three neurons at their preferred (fitted) orientation. The examples span approximately the full range of goodness of fits (i.e. R^2^ values) included in the analysis. (B) The fitted spatial receptive fields of the neurons shown in (A). (C) Response amplitudes of neurons shown in (A) to all orientations at preferred (fitted) position (unfilled markers), and fitted orientation tuning curves (blue lines).
